# Patient Engagement in Research Scale (PEIRS-22): Danish translation, applicability, and user experiences

**DOI:** 10.1186/s40900-023-00526-2

**Published:** 2023-12-07

**Authors:** Mille Guldager Christiansen, Helle Pappot, Clayon Hamilton, Trine Lund-Jacobsen, Anne Wilhøft Kristensen, Sara Nordentoft, Beverley Lim Høeg, Pernille Bidstrup, Iben Husted Nielsen, Jane Ulstrup, Anouk Kirsten Trip, Mary Jarden, Karin Piil

**Affiliations:** 1 Department of Oncology, Center for Cancer and Organ Diseases, Copenhagen University Hospital, Rigshospitalet, Denmark; 2 Faculty of Health and Medical Sciences, University of Copenhagen, Copenhagen, Denmark; 3 Department of Clinical Medicine, University of Copenhagen, Copenhagen, Denmark; 4 BC Mental Health and Substance Use Service at Provincial Health Service Authority, Simon Fraser University, British Columbia, Canada; 5 Department of Endocrinology, Center for Cancer and Organ Diseases, Copenhagen University Hospital, Rigshospitalet, Denmark; 6 Danish Centre for Particle Therapy, Aarhus University Hospital, Aarhus, Denmark; 7 Department of Neurosurgery, Center for Neuroscience, Copenhagen University Hospital, Rigshospitalet, Denmark; 8 Psychological Aspects of Cancer Research Group, Danish Cancer Society Research Center, Copenhagen, Denmark; 9 Institute of Psychology, University of Copenhagen, Copenhagen, Denmark; 10 Department of Hematology, Center for Cancer and Organ Diseases, Copenhagen University Hospital, Rigshospitalet, Denmark; 11 Copenhagen, Denmark; 12 Department of People and Technology, Roskilde University, Roskilde, Denmark

**Keywords:** Patient and Public Involvement, Patient engagement, Patient Engagement in Research Scale, Focus groups, PEIRS-22, Danish

## Abstract

**Background:**

Patient and Public Involvement (PPI) in health research is gaining increased attention and acceptance worldwide. Reliable measurements are crucial to accurately assess, monitor, and evaluate patient involvement efforts in research. The Patient Engagement in Research Scale (PEIRS-22) measures meaningful patient and family caregiver engagement in research. This study focuses on three primary objectives: (1) translation of the PEIRS-22 from English to Danish, followed by linguistic validation and cultural adaptation; (2) assessing the applicability of the Danish PEIRS-22; and (3) focus group interviews to explore the user experiences of PPI.

**Methods:**

A three-phase multi-method study was conducted. In phase one, the PEIRS-22 was translated, linguistically validated and culturally adapted to Danish. In phase two individuals from three distinct patient cancer advisory boards responded to the Danish version of PEIRS-22 to assess its applicability. Three focus group interviews were conducted in phase three, involving individuals from three patient cancer advisory boards.

**Results:**

The translation process resulted in a Danish version of PEIRS-22, conceptually and culturally equivalent to the English version. Overall, among individuals of the three advisory boards (*n* = 15) the applicability was found to be satisfactory, with no missing data and all items completed. The total PEIRS-22 score among the three advisory boards was 85.2 out of a possible 100, with higher scores indicating greater meaningful involvement. A nested sample of the three patient cancer advisory boards (*n* = 9) participated in focus group interviews. The analysis yielded four themes: (1) The Danish PEIRS-22 captured the intended cultural meaning and contributed to self-reflection, (2) Internal motivation is a driver for involvement (3), Involvement brought a personal sense of empowerment and (4) Meaningful involvement collaborations are fostered by a trustful atmosphere.

**Conclusions:**

The PEIRS-22 questionnaire has been translated, linguistically validated, and culturally adapted into Danish. We propose that the PEIRS-22 is now ready for use in Danish populations. This study provides a Danish version of the questionnaire that can be used to develop patient-centred practices and foster meaningful involvement and collaborations between patients and researchers in the field of cancer research in Denmark. Personal benefits of participating in PPI can vary, and we recommend using PEIRS-22 in conjunction with a qualitative approach to better explore perspectives on meaningful involvement.

***Trial registration*:**

The study was registered prospectively on October 22, 2022, by the Danish Data Protection Agency (jr. nr. P-2022–528).

**Supplementary Information:**

The online version contains supplementary material available at 10.1186/s40900-023-00526-2.

## Abstract (plain language summary)

Patient and public involvement (PPI) in research can improve research practices by ensuring that patients' voices are heard. Individuals’ lived experiences and unique viewpoints can contribute to refining research aims, ensuring they align with the needs and priorities of the target population. There is a growing interest in inviting patients into the research team as patient partners, for example, by establishing patient advisory boards. PPI can also involve caregivers and other stakeholders who are not usually thought of as members of the research team. For that reason, broadening our understanding of establishing meaningful PPI starts with measuring patient and family caregiver involvement. As such, the Patient Engagement In Research Scale (PEIRS-22) has been developed in the English language to measure meaningful patient and caregiver involvement. In this study, we aimed to (1) create a Danish version of the PEIRS-22 that respects any unique feature of Danish people, (2) assess the applicability of the Danish PEIRS-22, and (3) via focus-group interviews explore the user experiences of PPI. The patients and caregiver who were interviewed as part of the translation process expressed that the PEIRS-22 was easy to understand and captured the intended meanings. Fifteen other patient partners responded to the Danish version of PEIRS-22, and nine of them participated in the focus group interviews. One result was that creating a trusting and social atmosphere within the research group is important for promoting a personal sense of involvement.

**Supplementary Information:**

The online version contains supplementary material available at 10.1186/s40900-023-00526-2.

## Background

Patient and public involvement (PPI) in health research is gaining increased attention and acceptance worldwide. According to the National Institute for Health Research [1], patient and public involvement in research is defined as research conducted 'with' or 'by' members of the public rather than 'to', 'about', or 'for' them. Patients have a democratic right to participate in research about themselves [2] and due to their personal experiences, they provide valuable perspectives that advance the researchers' understanding [3, 4]. The democratic values of PPI, ensure that those affected by policies and research have an opportunity to participate and have their voices heard. Therefore, PPI is highly encouraged by patient organizations, funding agencies, scientific journals, and patient advocacy groups [2, 5]. Some of the benefits of involving patients in research may be improvements in study relevance, design, and recruiting procedures [3, 5]. However, since PPI is relatively novel it can present challenges and complexities. One challenge is tokenism, which occurs when the power imbalance between patients and healthcare professionals serves to achieve the professional or personal objectives of healthcare professionals rather than supporting the actual context [6, 7]. In line with this is symbolic inclusion, involving the inclusion of underrepresented groups to maintain a “diversity” balance in research, not necessarily because they are directly relevant to the research questions, but because they represent a specific group [8]. To prevent tokenism and symbolic inclusion, researchers should ensure that their involvement is both respectful and meaningful [8].

Traditionally, PPI has been centered on patients' contributions, while other users like families and informal caregivers are frequently being overlooked or dismissed as an extension of efforts to engage patients [9]. Effective patient and caregiver involvement necessitates an optimal environment to ensure that patients and caregivers can contribute effectively to key outcomes using their unique lived experiences [9].

PPI has mainly been integrated into the early phases of the research processes, with challenges and recommendations only briefly described [7]. PPI can present challenges and complexities. For instance, it can be time-consuming and demanding, especially when patients prioritize addressing their own unmet needs over participating in research. Additionally, conflicts may arise due to differing perspectives between patients and researchers [10]. In Denmark, the impact of PPI is still evolving and thus not well described [11], particularly among cancer populations. One way to involve patients in research is by including patients as members of patient advisory boards and as patient partners [12].

These challenges underscore the need for an active policy regarding the successful integration of high-quality PPI in cancer research. Improvements are needed to ensure meaningful involvement throughout the research cycle, as well as in evaluating and reporting its impact [5]. Further, identifying patients' motivations for involvement in research, allows researchers to design meaningful and engaging involvement opportunities that align with their interests [13]. This enhances patient engagement and increases the likelihood of active and sustained participation in research activities [12].

The quantitative evidence of the impact of PPI is limited and there is a gap regarding whether and how PPI improves health research quality [14, 15]. To address this gap, validated quantitative measures of PPI can be used to assess the effectiveness of the involvement method. By doing this it is possible to generate findings that can be more broadly applied contributing to an overall improvement in the quality of PPI [16]. A recent systematic review [17] identified 58 tools for measuring health-patient partnership outcomes and impact. Only a few of these tools were designed to measure both patients’ and caregivers’ involvement [17]. In the Danish context, there is a notable lack of such tools for measuring PPI.

The Patient Engagement in Research Scale (PEIRS) [16] has been developed to measure meaningful patient and family caregiver engagement. It originally included 37 items, but to reduce respondent burden it was shortened to a 22-item version (PEIRS-22) in 2021 [15]. In this scale, meaningful involvement in research is defined as the planned, encouraged, and valued involvement of patients and their surrogates, such as family caregivers [16]. PEIRS-22 is currently only published in English and has as far as we know never been tested for a cancer population. Thus, the accessibility of a Danish language version of the PEIRS-22 will establish the groundwork for measuring meaningful involvement and enable a broader application and comparison across studies and countries. Therefore, the aims of this study were to:

1. Translate the PEIRS-22 from English to Danish, followed by rigorous linguistic validation and cultural adaptation to capture the intended meanings and cultural nuances.
2. Assessing the applicability of the Danish PEIRS-22, providing a baseline for future measurements and comparisons
3. Explore the user experiences of PPI via focus group interviews.

The findings of the present study will offer valuable guidance for healthcare professionals, researchers, and policymakers in promoting effective patient engagement strategies, improving the relevance of research, and ultimately enhancing the experiences and outcomes of cancer patients within the Danish healthcare system.

## Methods and material

We used a multi-method study design consisting of three phases in accordance with our three research objectives. In phase one, patients were recruited for the cognitive debriefing interviews using purposeful sampling [18, 19] through an existing research group within a cancer patient organization and a caregiver advisory board. In phase two, convenience sampling [18, 19] was employed to recruit participants from three existing patient cancer advisory boards. The patient advisory boards represented individuals with lived experience of breast, gynecological, and brain cancer. The gynecological and breast cancer boards were recruited as research partners for a three-year PhD study, whereas the brain cancer board was recruited at the time of the development of the research protocol for a phase 1 clinical trial. The three boards participated as research partners from the projects inception, engaging in discussions about study design, outcomes, recruitment procedures, and interpretations of study results [11, 20].

In phases one and two the sample size was determined by information power as described by Malterud et al. [21]. In phase three, patients from phase two were invited to participate in focus group interviews. The inclusion criteria for the participants in all phases were ≥ 18 years of age and personal user experience in being involved in PPI. The Guidance for Reporting Involvement of Patients and the Public Checklist (GRIPP2)[22](short version) guided the reporting process (Additional file: 1).

### Phase 1: translation, linguistic validation, and cultural adaptation of PEIRS-22 from English to Danish

This phase followed the principles of good practice for the translation and cultural adaptation described in the International Society For Pharmacoeconomics and Outcomes Research (ISPOR) guideline [23]. This guideline recommends a ten-step procedure, from initial preparation to the final report. The ten steps used in our study are listed in Table 1, and each step is depicted in Additional file: 2.

**Table 1:** The 10-step process used to translate the PEIRS-22 into Danish

Steps	Methods	Outcomes
(1) Preparation	We obtained the necessary permissions and established a clinical expert group	Permission was granted by the developer of PEIRS-22, Dr. Hamilton Specialists in various cancer diagnoses (*n* = 11); professors (*n* = 2), senior researchers (*n* = 5), and doctoral students (*n* = 4) composed the clinical expert group
(2) Forward translation +( 3) Reconciliation	Key-in-country person and the project manager engaged a professional translator who initially translated PEIRS-22 from English to Danish	The three forward translations were reviewed and compared within the clinical expert group to identify discrepancies, and thus consensus translations were developed in the final reconciled forward translation. In a Danish context, the original English title proved to be too lengthy and general and was adjusted
(4) Back translation	Two additional translators being native speakers of English and fluent in the target language (Danish) translated the Danish version of PEIRS-22 into English	The two back-translated versions, as well as the translator's version, were intended to be conceptual rather than literal translations
(5) Back translation review	The original questionnaire and the two back translations were compared	Some phrases or words in English were found difficult to translate into Danish, for instance, “Convenience”, “Procedural requirements” and “Team Environment and Interaction” because no Danish words correspond to the original meaning. The wording in Danish was discussed until a consensus agreement was reached
(6) Harmonization	The clinical expert group compared the back-translated version against the original PEIRS-22 to examine discrepancies	The developer Dr. Hamilton was involved in clarifying wordings and meanings, for example, the alteration between “Research team members” and “The research project team”, after that we decided to use “research team members” consistently in the Danish version. Following discussions, the clinical expert group agreed on the revised Danish version to be ready for cognitive debriefing interviews
(7) Cognitive debriefing	An interview guide directed the semi-structured cognitive interviews Interviews were recorded and transcribed verbatim	A total of seven patients agreed to participate after two declined due to lack of time. The patient's characteristics are shown in Table 2, column 1. MGC conducted the interviews and at the beginning of the interviews, the think-aloud [21] procedure was introduced to the patients. The patients were encouraged to express their thoughts and considerations when responding to the items Overall, the patient partners valued the introduction to the questionnaire and found it easy to respond to. The patient partners highlighted that the items were understandable, however, they did not agree with the Danish words of convenience and the formulation of some of the items. The partners suggested adding a free text box. However, in the original questionnaire, this is not included and therefore it will not be a part of PEIRS-22. The patient partners expressed a suggestion to include a "not relevant" or "not applicable" option. However, after consulting with the clinical expert group and the scale's developer, Dr. Hamilton, we decided against it because it could interfere with the scale's ability to generate an interpretable total score
(8) Review of cognitive debriefing	The clinical expert group compared and discussed the comments and suggestions for improvement of the Danish version of PEIRS-22 to highlight and amend discrepancies	The clinical expert group discussed each item, considering the comments and perspectives shared by the patients. During this process, some of the suggestions were incorporated, while others remained unchanged due to their alignment with the original items. However, item FV3 asking: “I was offered sufficient recognition for my contributions (for example payment, authorship, or gifts)”, led to minor adjustments. The helping text was changed to: (for example catering, writing, lectures, or gifts). Any discrepancies were highlighted and discussed until the expert group reached a consensus Dr. Hamilton clarified wordings and meanings, for example, the alteration between “Research team members” and “The research project team”. Hence, we decided to use “research team members” consistently in the Danish version
(9) Finalization + 10) Proofreading	The Danish PEIRS-22 was checked for typographical and grammatical errors	Finally, word positions and choices were adjusted based on the findings to fit the questionnaire into Danish, and MCG and KP proofread and finalized the PEIRS-22. The research team then approved the final linguistically validated and equivalent Danish version (Additional file: 3)

### Phase 2: assessing the applicability of the Danish version of PEIRS-22

Patient partners from three Danish patient cancer advisory boards responded to the PEIRS-22. The data collection was conducted either on paper or electronically using REDCap (Research Electronic Data Capture)[24] based on the patient's preferences. Using a 5-point Likert scale, each item was translated to a numerical score (0–4) with 4 being highly agreed and 0 being strongly disagreed. The mean PEIRS-22 total score was calculated by taking the total sum score, dividing it by 88 (22 items _X_ 4 scores), and multiplying it by 100 [15]. As defined by Hamilton et al. [15], a total mean score of 92 or higher indicates extremely high meaningful involvement and a score of 70 or lower indicates a low to moderate level of meaningful involvement [25].

### Phase 3: focus group interviews exploring the user perspectives on PPI

Patient partners from the three advisory boards were invited to participate in the focus group interviews. One week prior to the interview the patient partners responded to the Danish PEIRS-22, and the moderators were provided with the group-level response data before conducting the focus group interviews. The Framework method as described by Gale et al. [25] was used to analyze the data and this method enables systematic analysis of homogeneous data using both an inductive and a deductive approach [25]. The interviews were guided by an interview guide (Additional file: 4) that included pre-defined sub-domains of meaningful patient involvement retrieved from PEIRS-22, and data was thus processed deductively.

## Results

A total of (*n* = 22) participants were recruited for phases one to three. Table 2 outlines the demographics.

**Table 2:** Demographics of the participants in the three study phases

Study phases	Phase 1 Translation process	Phase 2 Evaluation	Phase 3 Focus groups
Numbers (*n*)	7	15	9
**Age, mean years (range)**	56 (34–77)	60 (40–77)	61 (42–75)
	**N (Percent**)	**N (Percent**)	**N (Percent**)
*Gender*			
Women	4 (57)	12 (80)	6 (67)
Men	3 (43)	3 (20)	3 (33)
*Participants in the advisory boards*			
Patient	6 (86)	12 (80)	9 (100)
Caregiver	1 (14)	3 (20)	0
**The primary site of cancer**	N/A		
Brain		6 (40)	3 (33)
Breast		4 (27)	3 (33)
Gynecological		5 (33)	3 (33)
***Highest completed education***			
Basic school		3 (20)	2 (22)
High school or short-cycle tertiary	6 (86)	8 (53)	5 (55)
Higher education	1 (14)	4 (27)	2 (22)
*Employment status*			
Working (full or part-time)	1 (14)	7 (47)	4 (44)
Not working	6 (86)	8 (53)	5 (66)
Relationship status	N/A		
Married/living together		13 (87)	8 (89)
Single		2 (13)	1 (11)
**Years of involvement in the advisory board**	N/A		
< 6 months		1 (7)	
6 months to 1 year		11 (73)	8 (89)
1 year to 1.5 year		2 (13)	1 (11)
1.5 to 2 years		1 (7)	
**Participation in the advisory board (number of meetings)**	N/A		
1 to 3 meetings		5 (33)	3 (33)
3 to 6 meetings		8 (53)	4 (44)
6 to10 meetings		2 (13)	2 (22)

### Phase 1: translation, linguistic validation, and cultural adaption of PEIRS-22 from English to Danish

The results of the translation, linguistic validation, and cultural adaption process are outlined in Table 1 and Additional file: 2. In general, the patients believed that the Danish PEIRS-22 was relevant and easy to understand.

### Phase 2: applicability of the Danish version of PEIRS-22

A total of *n* = 15 patient partners (Table 2) were recruited and responded to the Danish PEIRS-22 questionnaire. The patient partners had a mean age of 60 years and were current patients/survivors (*n* = 12) or caregivers (*n* = 3). There were no missing data, and all items were completed, demonstrating the Danish PEIRS-22's overall good applicability.

The total mean score of PEIRS-22 was 85.2 (SD 14.4) out of a maximum of 100 (Table 3); 72.2 (SD 16.0) in the breast cancer patient advisory board, 89.3 (SD 13.8) in the gynecological cancer patient advisory board, and 90.1 (SD 10.2) in the brain cancer patient advisory board. The degree of meaningfulness was interpreted as described by Wang et al. [18], demonstrating that 70.1 is low, 70.1 to 82.7 is moderate, 82.7 to 92.0 is very meaningful, and 92.0 to 100 is extremely meaningful as outlined in Table 3.

**Table 3:** Total mean PEIRS-22 score per patient advisory board and interpretation

Patient advisory board	Number of patient partners	Mean PEIRS-22 total score (SD)	Interpretation of the summative PEIRS-22 score
Gynecological	5	89.3 (13.8)	Very meaningful
Breast	4	72.2 (16.0)	Moderately meaningful
Brain	6	90.1 (10.2)	Very meaningful
Overall in total	15	85.2 (14.4)	Very meaningful

### Phase 3: focus group interviews exploring the user experiences of PPI 

All 15 patient partners from the three advisory boards were invited to participate in the focus group interviews, but six declined to participate due to lack of time, resulting in the recruitment of a total of nine patient partners (Table 2). Three focus group interviews were conducted in separate diagnosis-specific groups. Interviews were conducted online via Microsoft Teams meetings in December 2022 and January 2023 and were audio-recorded and transcribed verbatim. Each interview lasted approximately one hour. KP moderated the interviews, while MGC co-moderated two interviews and HP co-moderated one interview. The moderators were experienced qualitative researchers and had no prior knowledge of the patient partners. The Framework method consists of the following seven steps [25] and data was analyzed by MCG and KP:

(1) Transcription. All transcripts were checked for inaccuracies by listening to each audio recording.; (2) Familiarization with the interview. To become familiar with the data, the transcripts were read several times; (3) Coding. MGC and KP individually coded the transcripts, highlighting informative sections; (4) Developing a working analytical framework. The analytical framework was formed after the coding was discussed and an agreement on a set of codes was reached; (5) Applying the analytical framework. MGC and KP went systematically through each transcript and selected codes from the analytical framework.; (6) Charting data into the framework matrix. All coding from the analytical framework was summarized; (7) Interpreting the data. The data was summarized and interpreted, and a comprehensive description of the user experiences was established. Four themes emerged from the focus group interviews: (1) The Danish PEIRS-22 captured the intended cultural meaning and contributed to self-reflection, (2) Internal motivation is a driver for involvement (3) Involvement brought a personal sense of empowerment, and (4) Meaningful involvement collaborations are fostered by a trustful atmosphere.

### Theme 1: the Danish PEIRS-22 captured the intended cultural meaning and contributed to self-reflection

The questionnaire was simple for the patient partners to understand and complete, and the topics were interesting and captured the overall essence of involvement. The level of experience that each of the three boards could endorse varied.

*“If you look at my response [to the questionnaire], you'll notice that the answers are right in the middle of neither agreeing nor disagreeing; just because I can't take a position on it right now doesn't mean I'm equal. It simply means I haven't decided yet.” *(Patient partner with breast cancer).

While for others, being a patient partner had been an extraordinarily and exceedingly gratifying experience, they noted that the overly optimistic interpretation of the response could potentially be misleading.

“When I had completed the questionnaire [PEIRS-22]; Since I am very satisfied, I was almost afraid to turn it in. Because the researcher may believe that there has hastily been placed some ticks. I am part of a very successful research project, so it was easy to be positive.” (Patient partner with brain cancer).

However, for some patient partners, it proved challenging to perceive themselves as partners. While they acknowledged their role in assisting, guiding, and contributing to the research, they did not personally identify themselves as patient partners.

“Somehow the questionnaire is difficult when you don't see yourself as a co-researcher because we haven't contributed that much yet.” (Patient partner with breast cancer).

For others, being a co-researcher was seen as positive and reinforcing and it provided them with a sense that their opinions were being actively explored and considered. This was associated with feelings of being valued and important.

“While answering the questionnaire, I was wondering if they were asking me this; if my opinion was important; and if I knew enough to answer this questionnaire. Then I realized that, yes, my opinion matters.” (Patient partner with gynecological cancer).

### Theme 2: internal motivation is a driver for involvement

The patient partners motivation to be involved in research originated from a desire to use their experiences for the benefit of others and thereby support the researchers in improving clinical practice for the benefit of future patients.

“If I could contribute to the researchers with my perspective in the research process, I thought it was important.” (Patient partner with brain cancer).

Some were motivated by the opportunity to learn about current and upcoming research. On the other hand, some partners were driven by the desire to prevent future patients from experiencing the negative experiences they had faced during their cancer treatment. Both motivations highlight the diverse reasons why patient partners choose to be involved in research. An example of this would be a traumatic experience that occurred during the pre-treatment stage. The patient partner shared, *“Nobody should go through what I went through, so I'd like to share my story with others.”* (Patient partner with gynecological cancer).

In contrast, some partners did not have a full understanding of what they had willingly agreed to participate in and be involved with.

“It is not what I expected; I thought we should contribute to what bothers patients, such as what they are dealing with and how they can cope with it.” (Patient partner with breast cancer).

Some patient partners emphasized the importance of improving their understanding of their involvement and the impact it had on the research project. They recognized that by gaining a deeper comprehension of their role, they could contribute more effectively and meaningfully to the research process.

“It is an inspiration that research points us in the right direction, but it is also important to understand how the words we say are received, and how our perspectives contribute to the future.” (Patient partner with brain cancer).

The patient partners motivation for being involved was viewed as voluntary, with no expectation of receiving gifts or financial compensation. They emphasized the significance of preserving the culture and premise of unpaid volunteer work in Denmark. As a result, the patient partners viewed the acknowledgment of their contributions through gestures such as mileage reimbursement for long-distance transportation or catering at meetings as thoughtful and appreciated acts.

“We get a sandwich, unlimited coffee, and transportation reimbursement. That is fine with me.” (Patient partner with brain cancer).

The patient partners made a variety of contributions both as research members and prior to meetings. These contributions included serving as discussants, offering feedback on written materials, educating healthcare professionals, and participating in peer-targeted videos. The number of time patient partners dedicated to these contributions was deemed acceptable as long as they could perceive the benefits of their efforts.

“I don't think it has been too much. I could easily have spent more time on the project. It appeals to me.” (Patient partner with gynecological cancer).

### Theme 3: involvement brought a personal sense of empowerment

For the patient partners, their involvement in research was of personal benefit because they felt a sense of personal growth as a result of the appreciation they received as valued members of the board. Among the patient partners with gynecological cancer, it was explained that their involvement made them honored, and they expressed a sense of pride in sharing their participation with others.

“Then people ask what your prerequisites [as a patient] are for joining a research group. Then I explain that I am an expert in my disease, and that is my unique contribution.” (Patient partner with gynecological cancer).

One of the benefits reported by the partners was the opportunity to connect with others who shared the same cancer diagnosis. They found solace in discovering that they had many similarities and experiences in common. The prospect of meeting each other at the meetings became something they looked forward to, and they expressed a willingness to provide support to one another if the need ever arose.

“I wouldn't hesitate for a second to reach out to someone if I needed it.” (Patient partner with brain cancer).

Among the breast and gynecological patient cancer advisory boards, the women had the desire to meet physically occasionally with the other patient partners. This occurred because they learned that they had many shared interests, and the numerous parallels strengthened their desire to meet with adequate time to freely chat.

“I believe we would benefit from meeting in person. The issue is that we are separated by a significant distance; hopefully, we will meet somewhere in between our homes.” (Patient partner with breast cancer).

Despite the seriousness of the topics discussed, the meetings had a positive atmosphere, as did the personal benefits of participating in research. The opportunity to contribute with their perspectives, insights, and lived experiences to the research team made them feel valued and heard. Some patient partners even stated that their participation and involvement with the research team made them courageous and empowered.

*“I believe that attending these meetings gives me more courage in life.”*

(Patient partner with gynecological cancer).

### Theme 4: meaningful involvement collaborations are fostered by a trustful atmosphere

The atmosphere and interaction were positive and based on mutual trust and confidentiality in the advisory boards. It was deemed crucial that the researcher generated trust in a warm, trustful, and accommodating manner.

“The researcher must be intuitive. When everyone knows each other, you're in the same situation, and you've talked a little, you feel safe.” (Patient partner with gynecological cancer).

The atmosphere among the patient partners was characterized by respect and recognition for each other’s diverse backgrounds and personal circumstances.

“When you are diagnosed with a life-threatening disease, something changes in your mind and acceptance of others' challenges.” (Patient partner with brain cancer).

Overall, the researchers' facilitation, prioritization, involvement, and leadership skills, as well as their clinical and personal qualities, were highly valued by the patient partners. Some of the structural elements that patient partners appreciated were the overall status of the project's momentum, highlighting the patient partners' contribution to the project, and persistence. Overall, it was crucial for the partners to feel acknowledged and understood by the researchers. They emphasized the importance of having dedicated time and space to explain their unique cancer situation, concerns, and needs.

“We begin meetings by recapitulating; where we are, without going into too much detail, but with respect for where we are.” (Patient partner with brain cancer).

The patient partners expressed a welcoming attitude towards the inclusion of new members if the researchers believed their involvement could bring additional value to the research project. However, one concern raised by the partners was the potential for the patient advisory board to become too large, which could make it challenging for individual members to actively contribute and engage in discussions.

“If the group grows significantly, there will be too little time and room for all to have a say.” (Patient partner with brain cancer).

Not a lot of new [patients]. They could bring something new to the table, as we have seen many of the same things.” (Patient partner with gynecological cancer).

Involving caregivers in the patient advisory board for brain cancer was an inclusive approach, considering the significant role these informal caregivers play in the lives of patients with brain cancers. The caregiver's contribution was valued and appreciated due to their ability to effectively communicate their experiences and knowledge with peers at the advisory board meetings.

“Well, it makes us talk about things together.” (Patient partner with brain cancer).

## Discussion

Overall, the translation process, which involved cognitive interviews, demonstrated the usefulness and applicability of the questionnaire, although certain suggestions for improving the comprehension of the items were identified. PEIRS-22 includes seven domains of meaningful involvement in research (procedural requirements, convenience, contributions, team environment & interaction, support, feeling valued, and benefits) [15]. These domains were found relevant for PPI in a Danish context. We propose that the PEIRS-22 questionnaire is now ready for use in Danish populations to measure the degree of meaningful involvement [26]. The linguistic validation and cultural adaptation of PEIRS-22 was, in general, comparable to the original, even though minor adjustments were made. In the cognitive interviews, patient partners provided alternate wordings for some items, but these could not be changed without affecting the original and intended meaning. The patient partners explicitly stated that they had understood the items in their entirety, demonstrating the PEIRS-22 cultural relevance, appropriateness, and comprehension in Danish.

A Danish version of PEIRS-22 has now been used for the first time in a cancer population as well as in Danish. Our study's findings revealed a mean score of 85.2, which is comparable to the means identified among the other populations. A stakeholder committee in respiratory disease (*n* = 15) had a mean PEIRS-22 score of 79.83 [27], and stakeholders with Down syndrome (*n* = 22) [28] demonstrated a mean PEIRS-22 score of 93.5. Due to the small sample size, it is generally challenging to generalize the findings. In our study, which was conducted in a context where PPI is gaining momentum, we purposefully chose a sample for evaluation that represented a wide range of cancer diagnoses and patient advisory boards. The results of PEIRS-22 showed that the patient partners overall felt very meaningfully involved, as indicated by the overall mean score (85.2).

We found that some patient partners and family caregivers felt unfamiliar with being considered patient partners. One reason could be that they do not see themselves as equal or valued members of the research team, because they are not professional researchers. This is an interesting finding, as this component is an important parameter of being meaningfully involved [29]. As we find, a researcher facilitating a patient advisory board should initially conduct an alignment of mutual expectations and provide explanations of the wordings used. The researcher must be capable of leading and facilitating, and roles and expectations must be clearly defined; otherwise, the patient partner may become frustrated or even uninterested [30]. We recommend that the researcher be specific in expressing the ongoing impact of the involvement and acknowledge the efforts of the patient partners. A further consideration is how the involvement approach affects the experience of meaningful involvement of patient/family caregiver partners. In this context, PEIRS-22 is valuable, because only by involving patients will we be able to assess whether PPI affects the relevance, appropriateness, and usefulness of research. Furthermore, the cultural context in which researchers and patient partners work could potentially have an impact on PPI. We propose conducting additional research on PPI in a Danish context to further explore and expand upon the findings of the current study.

In some countries, patient partners are compensated with gifts or money [31, 32], whereas in others, such as Denmark, contributions are mostly voluntary, which can result in a variety of incentives [33]. Our findings highlight the willingness of patient partners to participate in research even when their contributions are not explicitly monetarily acknowledged, suggesting that their motivation for involvement may stem from altruistic reasons. The patient partners expressed a strong sense of dedication and motivation to share their experiences, driven by the desire to assist future patients in similar situations. This finding can be comprehended in the context of the Danish welfare model, which is characterized by high-income equality, universal access to healthcare, and modest financial compensation for income loss due to illness or disability [34]. Despite the patient partners primary motivation being altruistic, they also expressed appreciation for reimbursement of transportation costs and small gifts. As the field of PPI continues to expand, it is anticipated that researchers will increasingly seek funding that includes provisions for compensating partners for their valuable contributions as patient partners. Steadily, one of the items in PEIRS-22 [15] asks if patient partners receive sufficient recognition (payment, authorship, or gifts) and Danish partners expressed that the practice of compensating patient partners for their involvement, as mentioned in the item, was unsuitable within the Danish cultural context. Consequently, the wording of the item was retained and the examples provided in parentheses were modified to align with Danish cultural norms.

Our qualitative findings are generally in line with Lauzon-Schnittka et al. [4], who investigated the perspectives of patient partners with a broad range of diseases. Our findings show that, despite having a history of being a cancer patient, they are willing to be involved, implying that involvement allows individuals to grow personally. This is in concordance with the results by Hovén et al. [30] who discovered that taking part in research activities can have therapeutic benefits as well as a sense of usefulness and enjoyment. Another finding from the focus groups in our study was that being a patient partner can be an extraordinarily and exceedingly gratifying experience, which led to overly positive questionnaire responses. It is important to acknowledge the potential influence of social desirability bias [35] on the patient partners responses to the PEIRS-22 questionnaire. Given this possibility, we cannot definitively conclude that our results are entirely unaffected by such bias [35]. To gain a clearer understanding, further research is necessary to investigate whether ceiling and floor effects are present when individuals respond to the questionnaire. This will help to ensure the accuracy and reliability of the results [36].

There is a lack of understanding of the challenges of involving patient cancer partners in research [37], and their potential involvement may lead to tokenistic involvement or symbolic inclusion [8, 38]. The majority of structured evaluation tools have been developed using unpublished, project-specific instruments, which limits the potential for comparison and mutual learning across involvement research projects [38]. PEIRS-22 may assist researchers in determining which areas need improvement and which areas are performing well and hereby it can function as an overall evaluation tool [15]. Further, this underscores the need for guidelines that provide advice on the measurement of meaningful PPI, so that researchers can determine whether they have used PPI appropriately [39]. Furthermore, advances in the description and evaluation of PPI have an impact on understanding and evaluating the benefits and challenges of PPI in cancer research [39].

Finally, more knowledge is needed about the involvement of caregivers, as it is unknown whether advisory boards solely with patients are better than mixed advisory boards including both patients and caregivers. Further investigation is required to understand the potential benefits and advantages of the establishment of patient advisory boards, ensuring that the most effective and inclusive methods of engagement are utilized. Caregivers in our study took part in cognitive interviews and the response to PEIRS-22 but were hindered from participating in the focus group interviews, so their perspectives have yet to be explored. Nevertheless, in a study among patients with chronic kidney disease and their caregivers [40], it was identified that they struggled with a high caregiver burden and that it limited their ability to contribute to research. Further, patients and caregivers believed that the limited availability of opportunities to engage in involvement activities, coupled with the need to actively seek out these opportunities, often at personal costs, posed challenges to achieving inclusion and diversity [40]. This underscores the need for a deeper understanding of caregiver involvement in PPI, especially among cancer populations. Moreover, the perspectives of the researchers must be examined for future research to expand the application of PPI and promote patient advisory boards. This is highlighted in a recent study among doctoral students in Europe [37], identifying that there is a need to improve doctoral student's knowledge and skills through structured training to help them incorporate PPI into their research projects.

### Strengths and limitations

The Danish translation, cultural adaptation, and linguistic validation of the PEIRS-22 followed the ISPOR guidelines [23], which secures a systematic approach. The multidisciplinary clinical expert group provided broad perspectives, which strengthened the discussions and critical interpretation of the results. One patient partner qualified the findings and became a co-author, which is a strength due to the inclusion of the patient perspective; however, recognizing that no patients or caregiver partners were involved in the design phase of this study is a limitation. Dr. Hamilton, the developer of the PEIRS-22, was a part of the author group and consulted on areas of the questionnaire's understanding, which contributes to the questionnaire's overall concept clarity and usability. The patient partners in this study had different cancer diagnoses or were caregivers, which increases the study's strength and generalizability.

The inclusion of caregivers in the cognitive interviews and pilot testing is a strength, however, their absence in the focus group interviews is a limitation. There is a need for further investigation into the level of effort required for PPI, as well as the potential risks of unbalanced power dynamics where stakeholders are included symbolically or nominally without genuine opportunities for meaningful contribution [8, 12]. More information is needed to investigate various aspects, including the time consumption of researchers, their thoughts, and reflections on facilitating advisory boards. This research would provide valuable insights into how to effectively facilitate and optimize the functioning of advisory boards, ensuring that researchers are adequately supported and able to engage meaningfully with the input and feedback received from patient partners.

## Conclusion

The PEIRS-22 questionnaire has been translated, linguistically validated, and culturally adapted into Danish and is now ready for use. The Danish PEIRS-22 was found simple to complete, and the domains in the questionnaire were found relevant. Our results showed that the patient partners generally experienced very meaningful involvement. This study reveals that among patient partners, the internal motivation for involvement in cancer research stems from an altruistic desire and an enthusiasm for developing a clinical practice that will benefit future patients. The findings contribute to developing patient-centred research practices by quantifying the user experience in the field of cancer research in Denmark. Finally, individual perceived benefits can vary, and we recommend using PEIRS-22 in conjunction with a qualitative approach to better explore perspectives on meaningful involvement.

### Supplementary Information

**Additional file 1.** GRIPP2 reporting checklist.

**Additional file 2.** Linguistic translation and validation of PEIRS-22.

**Additional file 3.** The Danish version of PEIRS-22.

**Additional file 4.** Guide for focus group interviews.

## Data Availability

The datasets used and/or analyzed during the current study are available from the corresponding author upon reasonable request.

## Abbreviations

PPI: Patient and public involvement
PEIRS-22: The Patient Engagement in Research Scale (22 items)
ISPOR: International Society For Pharmacoeconomics and Outcomes Research
KP: Karin Piil
MGC: Mille Guldager Christiansen
HP: Helle Pappot
