# Supplementary material for: Patient Engagement in Research Scale (PEIRS-22): Danish translation, applicability, and user experiences
Source: Res Involv Engagem. 2023 Dec 7;9:115. doi: 10.1186/s40900-023-00526-2 (PMC10704757; doi:10.1186/s40900-023-00526-2)
Supplement: Supplementary file 1 — Additional file 1. GRIPP2 reporting checklist. [file 40900_2023_526_MOESM1_ESM.docx]

GRIPP2 reporting checklists [1]

| Section and topic | Item | Reported on page No |
| --- | --- | --- |
| 1: Aim | Report the aim of PPI in the study | 3 |
| 2: Methods | Provide a clear description of the methods used for PPI in the study | 3-6 |
| 3: Study results | Outcomes—Report the results of PPI in the study, including both positive and negative outcomes | 6-10 |
| 4: Discussion and conclusions | Outcomes —Comment on the extent to which PPI influenced the study overall. Describe the positive and negative effects | 10-11 |
| 5: Reflections/critical perspective | Comment critically on the study, reflecting on the things that went well and those that did not, so others can learn from this experience | 11-12 |

PPI = patient and public involvement

[1] S. Staniszewska *et al.*, ‘GRIPP2 reporting checklists: Tools to improve reporting of patient and public involvement in research’, *BMJ*, vol. 358, 2017, doi: 10.1136/bmj.j3453.
