# Supplementary material for: Patient Engagement in Research Scale (PEIRS-22): Danish translation, applicability, and user experiences
Source: Res Involv Engagem. 2023 Dec 7;9:115. doi: 10.1186/s40900-023-00526-2 (PMC10704757; doi:10.1186/s40900-023-00526-2)
Supplement: Supplementary file 2 — Additional file 2. Linguistic translation and validation of PEIRS-22. [file 40900_2023_526_MOESM2_ESM.docx]

| **Original version PEIRS -22** | **Translation English to Danish**  **Project manager** | **Translation English to Danish**  **Professional 1** | **Translation English to Danish**  **Key-in-country-person** | **Reconciliation meeting** | **Back translation (Danish to English)**  **Professional 2** | **Back translation (Danish to English)**  **Professional 3** | **Points to consider**  **Back translation review** | **Harmonization** | **Cognitive debriefing** | **Review cognitive debriefing** | **Proof reading/finalization** |
| --- | --- | --- | --- | --- | --- | --- | --- | --- | --- | --- | --- |
| **Title:** Patient Engagement In Research Scale – PEIRS -22 | Patient involvering I forskning – PEIRS-22 | % | Oplevelsen af Patient Involvering i Forskningsprojekt – DANISH PEIRS-22 | Involvering af patienter og pårørende i forskningsprojekter  Dansk version af PEIRS-22 | Involvement of patients and relatives in research projects PEIRS-22 | Participation of patients and relatives in research projects | Involvement/participation/engagement | Involvering af patienter og pårørende i forskningsprojekter  Dansk version af PEIRS-22 | Ordet inddragelse – mere positivt  Forslag titel;  InddragelseI forskningsprojekter | Titel fastholdes:  Involvering af patienter og pårørende i forskningsprojekter  Dansk version af PEIRS-22 |  |

| **Original version PEIRS -22** | **Translation English to Danish**  **Project manager** | **Translation English to Danish**  **Professional 1** | **Danish translation**  **Key-in-country-person** | **Reconciliation meeting** | **Back translation**  **Professional 2** | **Back translation**  **Professional 3** | **Points to consider**  **Back translation review** | **Harmonizaton** | **Cognitive debriefing** | **Review cognitive debriefing** | **Proof reading/finalization** |
| --- | --- | --- | --- | --- | --- | --- | --- | --- | --- | --- | --- |
| **INSTRUCTIONS:** Thinking about your experience as a patient partner in the project, please respond to the statements by choosing only one box for each statement. If you are unsure about which option to choose for a statement, please give the best response you can. This questionnaire may take you about 3 to 7 minutes to complete. |  | **VEJLEDNING:** Tænk på din oplevelse som patientpartner i projektet og svar på udsagnene ved at vælge ét afkrydsningsfelt for hvert udsagn. Hvis du ikke er sikker på, hvilken valgmulighed du skal vælge for et udsagn, skal du give det bedste svar, du kan. Det tager ca. 3 til 7 minutter at udfylde dette spørgeskema |  | **Instruktioner:** Tænk på din oplevelse med at være involveret i forskningsprojektet og sæt venligst kun ét kryds i boksen ved hvert udsagn. Hvis du er usikker på hvilket udsagn du skal vælge, så vælg det udsagn der falder dig først ind.  Det vil tage dig 3 til 7 minutter at besvare dette spørgeskema.    **Definition Forskergruppe:** Med forskergruppen menes alle involverede i forskningsprojektet, det vil sige patienter, pårørende og forskere som udgør en samlet gruppe. | **Instructions:** Consider your experience of being part of a research group and being involved in the research project and then please tick just one box under each statement. If you are unsure which box to tick, choose the response that occurs to you spontaneously. The questionnaire takes about three to seven minutes to complete. | **Instructions:** Reflect on your experience of being a part of the research group and your participation in the research project, and set one cross only in the box by each response. If you are unsure which response you should choose, choose the response that comes to your mind first.  It will take you about 3 to 7 minutes to answer the questionnaire. | Godkende instruktioner | **Instruktioner:** Tænk på din oplevelse med at være involveret i forskningsprojektet og sæt venligst kun ét kryds i boksen ved hvert udsagn. Hvis du er usikker på hvilket udsagn du skal vælge, så vælg det udsagn der falder dig først ind.  Det vil tage dig 3 til 7 minutter at besvare dette spørgeskema.  **Definition forskergruppe:** Med forskergruppen menes alle involverede i forskningsprojektet, det vil sige patienter, pårørende og forskere som udgør en samlet gruppe.  **Definition projekt:** Med projekt menes de opgaver som skal gennemføres i en given kontekst og periode | Anvende dots for bedre overblik  Skrive hvor mange spørgsmål der er  Gør det så nemt som muligt  Indsætte en ikke relevant boks  Indsætte projektgruppe også: Projektgruppe  Med projekt menes de opgaver som skal gennemføres i denne sammenhæng | Enighed om ingen danske defintioner,  :Forsknings projektgruppe  Anvendes konsistent igennem hele skemaet |  |

| **Original** | Strongly Agree | Agree | Neutral | Disagree | Strongly Disagree |
| --- | --- | --- | --- | --- | --- |
| **English to Danish**  **(project manager, Professional 1, and Key-in-contry-person)** | Meget enig  ☐ | Enig  ☐ | Neutral  ☐ | Uenig  ☐ | Meget uenig  ☐ |
| **Back translation, Professional 2** | Fully agree | Agree | Neutral | Disagree | Fully disagree |
| **Back translation, Professional 3** | Agree completely | Agree | Neutral | Disagree | Disagree completely |

| **Original version PEIRS -22** | **Translation English to Danish**  **Project manager** | **Translation English to Danish**  **Professional 2** | **Translation English to Danish**  **Key-in-contry-person** | **Reconciliation meeting** | **Back translation (fra dansk til eng)** | **Back translation (fra dansk til eng)** | **Opmærksomheds punkter**  **Back translation review** | **Harmonization** | **cognitive debriefing** | **Cognitive debriefiing review** | **Proof reading/finalization** |
| --- | --- | --- | --- | --- | --- | --- | --- | --- | --- | --- | --- |

| **Procedural Requirements** | **Generelt om projektet** | **Proceduremæssige krav** | **Generelt om projektet** | **Generelt om projektet** | **General aspects of the research project** | **About the research project generally** | Adskiller sig fra original | **Generelt om projektet** | **Generelt om projektet** | Ingen ændringer |  |
| --- | --- | --- | --- | --- | --- | --- | --- | --- | --- | --- | --- |
| The following seven (7) statements are about your general experiences throughout the project. | De følgende syv (7) spørgsmål omhandler dine generelle oplevelser gennem hele projektet | De følgende syv (7) udsagn handler om dine generelle oplevelser gennem hele projektet. | De følgende syv (7) udsagn handler om dine generelle oplevelser gennem hele projektet | De følgende syv (7) udsagn handler om dine generelle oplevelser **gennem hele** forskningsprojektet | The following seven (7) statements relate to your general experiences throughout the research project | The following seven (7) statements are about your general experiences throughout the whole research project | Hele – ud  I istedet for gennem? | De følgende syv (7) udsagn handler om dine generelle oplevelser gennem hele projektet | Klart og tydeligt | Klart og tydeligt |  |
| PR2. The research team members were properly introduced to each other | Medlemmerne af forskningsgruppen/teamet blev introduceret til hinanden på en ordentlig måde | PR2. Forskningsteamets medlemmer blev præsenteret ordentligt for hinanden | PR2. Forskergruppens medlemmer blev ordentligt introduceret til hinanden | Forskergruppens medlemmer blev ordentligt  introduceret til hinanden | The members of the research group were introduced to each other in a proper fashion | The members of the research were properly introduced to each other | Meningen er den samme, men ordlyden er forskellig | Forskergruppens medlemmer blev ordentligt  introduceret til hinanden | Medlemmerne i projektgruppen blev ordentligt introduceret til hinanden | Forskningsprojekt gruppens medlemmer blev ordentligt introduceret til hinanden | Forskningsprojekt gruppens medlemmer blev ordentligt introduceret til hinanden |
| PR9. In general, I had sufficient opportunities to contribute to the project | Generelt havde jeg tilstrækkelige muligheder for at bidrage til projektet | PR9. Jeg havde generelt tilstrækkelige muligheder for at bidrage til projektet | PR9. Generelt havde jeg tilstrækkelige muligheder for at bidrage til projektet | Jeg havde generelt tilstrækkelige muligheder for at bidrage til forskningsprojektet | I generally had ample opportunity to contribute to the research project | In general I had sufficient opportunities for contributing to the research project | Ample/sufficient | Jeg havde generelt tilstrækkelige muligheder for at bidrage til projektet | Jeg havde generelt mulighed for at bidrage til projektet | Jeg havde generelt muligheder for at bidrage til projektet | Jeg havde generelt muligheder for at bidrage til projektet |
| PR10.I was able to perform my tasks for the project | Jeg var I stand til at udføre mine opgaver for projektet | PR10. Jeg var i stand til at udføre mine opgaver for projektet | PR10. Jeg var i stand til at udføre mine opgaver for projektet | Jeg var i stand til at udføre mine opgaver for forskningsprojektet | I was able to carry out my activities for the research project | I was able to perform my assignments for the research project | Carry out/perform  Activities/assignments | Jeg var i stand til at udføre mine opgaver i projektet | Jeg havde tilstrækkelige forudsætninger for at bidrage til projektet? | Jeg var i stand til at udføre mine opgaver i projektet | Jeg var i stand til at udføre mine opgaver i projektet |
| PR11. I participated in making decisions about the project | Jeg deltog i at træffe beslutninger om projektet | PR11. Jeg var med til at træffe beslutninger om projektet | PR11. Jeg var med til at træffe beslutninger om projektet | Jeg var med til at træffe beslutninger om forskningsprojektet | I was involved in decision-making about the research project | I helped make decisions about the research project | Involved/helped  Decisionmaking/  decisions | Jeg var med til at træffe beslutninger om projektet | Hvad hvis det ikke var hensigten at man skal træffe beslutninger? | Jeg var med til at træffe beslutninger om projektet | Jeg var med til at træffe beslutninger om projektet |
| PR12. I received sufficient updates about the project | Jeg modtog tilstrækkelige opdateringer om projektet | PR12. Jeg fik tilstrækkelige opdateringer om projektet | PR12. Jeg modtog tilstrækkelige opdateringer om projektet | Jeg modtog tilstrækkelige opdateringer om forskningsprojektet | I was kept properly up to date about the research project | I received sufficient updates about the research project | Kept properly/received | Jeg modtog tilstrækkelige opdateringer om projektet | Tilstrækkelige – nok  Tilfredsstillende  Jeg blev holdt orienteret om projektet  Jeg blev løbende orienteret om projekter | Jeg modtog tilstrækkelige opdateringer om projektet | Jeg modtog tilstrækkelige opdateringer om projektet |
| PR13.Communication within the research team was clear throughout the project | Kommunikationen med forskergruppen var tydelig igennem hele projektet (perioden) | PR13. Kommunikationen i forskningsteamet var tydelig gennem hele projektet | PR13. Kommunikationen i forskergruppen var tydelig gennem hele projektet | Kommunikationen i forskergruppen var tydelig i forskningsprojektet | Communication in the research group was transparent throughout the research project | Communications in the research group were clear throughout the whole research project | Transparent/clear | Kommunikationen i forskergruppen var tydelig gennem hele projektet | Alternativer  Føler du at kommunikationenen i projektgruppen var forståelig gennem hele projektet?  Kommunikationen i forskergruppen var tydelig gennem hele projektet.  Jeg oplevede at kommunikationen i projektgruppen var forståelig gennem hele projektet | Kommunikationen i forskningsprojekt gruppen var tydelig gennem hele projektet | Kommunikationen i forskningsprojekt gruppen var tydelig gennem hele projektet |
| PR14. The project was worth the time I spent on it | Projektet var værd at bruge tid på | PR14. Projektet var den tid værd, som jeg brugte på det. | PR14.  Projektet var den tid værd, jeg brugte på det | Projektet var den tid værd, som jeg brugte på det. | The research project was worth the time I spent on it | The research project was worth the time I spent on it | ens | Projektet var den tid værd, som jeg brugte på det. | Generelt – ikke et godt spørgsmål.  Jeg føler jeg har brugt min tid rigtigt  Jeg har brugt min tid rigtigt ved at være med i projektet | Projektet var værd at bruge min tid på | Projektet var værd at bruge min tid på |

| **Convenience** | **Praktik/behagelig** | **Praktikalitet** | **Bekvemmelighed** | **Bekvemmelighed** | **Ease** | **Convenience** | Ease/convenience | Belejlighed | Ikke godt  Udbytte  Bekvemmelighed/nemhed/komfortabel  **Belejligt** | Belejligt | Belejligt |
| --- | --- | --- | --- | --- | --- | --- | --- | --- | --- | --- | --- |
| The following three (3) statements are about how convenient it was for you to contribute throughout the project. | De følgende tre udsagn omhandler, hvor behageligt det var for dig at bidrage gennem hele projektet. | De følgende tre (3) udsagn handler om, hvor praktisk det var for dig at bidrage gennem hele projektet. | De følgende tre (3) udsagn handler om, hvor praktisk det var for dig at bidrage gennem hele projektet. | De følgende tre (3) udsagn handler om, hvor bekvemt det var for dig at bidrage i forskningsprojektet. | The following three (3) statements relate to the ease with which you were able to contribute throughout the research project | The following three (3) statements are about how convenient it was for you to contribute throughout the whole research project | relate to the ease/contribute | De følgende tre (3) udsagn handler om, hvor belejligt det var for dig at bidrage gennem hele projektet | Klart og tydeligt |  |  |
| CN1.I had the opportunity to provide input into selecting my tasks for the project | Jeg havde mulighed for at give input til udvælgelsen af mine opgaver til projektet | CN1. Jeg havde mulighed for at være med til at udvælge mine opgaver for projektet | CN1.  Jeg havde mulighed for at komme med input til udvælgelsen af ​​mine opgaver til projektet | Jeg havde mulighed for at være med til at udvælge mine opgaver i forskningsprojektet | I was the opportunity to be involved in selecting my activities for the research project | I had the opportunity to help choose my assignments for the research project | To be involved/  Activities/choose  Assignments/activities | Jeg havde mulighed for at være med til at udvælge mine opgaver i projektet | Hvad hvis ikke meningen?  Jeg havde mulighed for at vælge mine opgaver i projektet | Jeg havde mulighed for at være med til at vælge mine opgaver i projektet | Jeg havde mulighed for at være med til at vælge mine opgaver i projektet |
| CN3.Throughout the project, I had sufficient time to complete my tasks for the project | Gennem hele projektet havde jeg tilstrækkelig tid til at udføre mine opgaver for projektet | CN3. Under hele projektet havde jeg nok tid til at udføre mine opgaver for projektet | CN3.  Gennem hele projektet havde jeg tilstrækkelig tid til at udføre mine opgaver for projektet* | Jeg havde tilstrækkelig tid til at udføre mine opgaver gennem hele projektet | I had sufficient time to carry out my activities throughout the research project | I had sufficient time to perform my assignments throughout the whole research project | % throughout start  time to carry out my activities  time to perform my assignments  Gennem hele erstattes med i | Jeg havde tilstrækkelig tid til at udføre mine opgaver gennem hele projektet | Tid i forhold til hvad?  Jeg havde mulighed for at udføre mine opgaver gennem hele projektet | Jeg havde tilstrækkelig tid til at udføre mine opgaver gennem hele projektet | Jeg havde tilstrækkelig tid til at udføre mine opgaver gennem hele projektet |
| CN4.I had opportunities to express my views | Jeg havde mulighed for at udtrykke mine synspunkter | CN4. Jeg havde lejlighed til at udtrykke mine synspunkter | CN4.  Jeg havde mulighed for at udtrykke mine synspunkter | Jeg havde mulighed for at udtrykke mine synspunkter | I had the opportunity to express my points of view | I had the opportunity to express my views | my points | Jeg havde mulighed for at udtrykke mine synspunkter | Jeg havde mulighed for at udtrykke mine synspunkter | Jeg havde mulighed for at udtrykke mine synspunkter | Jeg havde mulighed for at udtrykke mine synspunkter |

| **Contributions** | **Bidrag** | **Bidrag** | **Bidrag** | **Bidrag** | **Input** | **Contributions** | Input/ Contributions | **Bidrag** |  | Bidrag |  |
| --- | --- | --- | --- | --- | --- | --- | --- | --- | --- | --- | --- |
| The following three (3) statements are about your contributions throughout the project. | De følgende tre udsagn omhandler dit bidrag gennem hele projektet. | De følgende tre (3) udsagn handler om dine bidrag gennem hele projektet. | De følgende tre (3) udsagn handler om dine bidrag gennem hele projektet. | De følgende tre (3) udsagn handler om dine bidrage i projektet. | The following three (3) statements relate to your input throughout the research project | The following three (3) statements are about your contributions throughout the whole research project | relate to/your contribution | De følgende tre (3) udsagn handler om dine bidrag gennem hele projektet | De følgende tre (3) udsagn handler om dine bidrag gennem hele projektet |  |  |
| CT1. I contributed by providing my perspective | Jeg bidrog ved at give mit perspektiv | CT1. Jeg bidrog ved at komme med mit perspektiv | CT1.  Jeg bidrog ved at give mit perspektiv | Jeg bidrog ved at give mit perspektiv | I contributed to the research project by sharing my perspective | I contributed to the research project by giving my perspective | Sharing/by giving | Jeg bidrog til projektet ved at give mit perspektiv | Synspunkt/mening  Jeg bidrog til projektet ved at sige min mening | Jeg bidrog til projektet ved at give mit perspektiv | Jeg bidrog til projektet ved at give mit perspektiv |
| CT2.My contributions were a good use of my time | Mit bidrag var en god brug af min tid | CT2. Mine bidrag var en god anvendelse af min tid | CT2.  Mine bidrag var en god udnyttelse af min tid | Det var en god udnyttelse af min tid at bidrage | My input into the project felt like time well spent | It was worth my time to contribute | Helt forskellige | Det var en god udnyttelse af min tid at bidrage  **Mine bidrag var en god brug af min tid** | Gentagelse PR14 (tid)  Dårlig formulering  Det var tiden værd at bidrage  Det var en god brug af min tid at bidrage  At bidrage, var en god brug af min tid | Det var en god brug af min tid at bidrage | Det var en god brug af min tid at bidrage |
| CT4. My workload in the project was manageable | Min arbejdsbyrde i projektet var overskuelig | CT4.  Min arbejdsbyrde i projektet var overskuelig | CT4.  Min arbejdsbyrde i projektet var overskuelig | Min arbejdsbyrde i forskningsprojektet var overskuelig | My workload in the research project was manageable | My assignments for the research project were manageable | assignments | Mine arbejdsopgaver i projektet var overskuelig | Mine opgaver i projektet var overskuelige | Mine opgaver i projektet var overskuelige | Mine opgaver i projektet var overskuelige |

| **Team Environment and Interaction** | **Arbejdsmiljø og samspil i teamet** | **Teammiljø og -interaktion** | **Teammiljø og interaktion** | **Miljø og samspil i forsker-gruppen-**  **Samarbejde i forskergruppen** | **Working climate and interaction within the research group** | **Environment and interaction in the research group** |  | **Samarbejde i forskergruppen** | Samarbejde i projektgruppen  Eller  samarbejde | Samarbejde i forskningsprojekt  gruppen | Samarbejde i forskningsprojekt  gruppen |
| --- | --- | --- | --- | --- | --- | --- | --- | --- | --- | --- | --- |
| The following two (2) statements are about the research environment and interaction throughout the project | De følgende to udsagn handler om forskningsmiljø og samspil gennem hele projektet | De følgende to (2) udsagn handler om forskningsmiljøet og interaktionen gennem hele projektet | De følgende to (2) udsagn handler om forskningsmiljøet og samspillet gennem hele projektet | De følgende to (2) udsagn handler om miljø og samspil i forskningsprojektet | The following two (2) statements relate to the working climate and interaction within the research group throughout the research project | The following two (2) statements are about the environment and the interaction in the research group throughout the whole of the research project |  | De følgende to (2) udsagn handler om samarbejdet i forskergruppen gennem hele projektet |  |  |  |
| T2. I was an equal partner in the research project team | Jeg var en ligeværdig partner i projektets forskningsgruppe | T2. Jeg var en ligeværdig partner i forskningsprojektteamet | T2.  Jeg var en ligeværdig partner i forskningsprojektgruppen | Jeg var en ligeværdig partner i forskergruppen | I was an equal partner in the research group | I was an equal partner in the research group | ens | Jeg var en ligeværdig partner i forsknings projekt gruppen | Er det meningen?  Jeg var en værdsat partner i projektgruppen | Jeg var en ligeværdig partner i forsknings projekt gruppen | Jeg var en ligeværdig partner i forsknings projekt gruppen |
| T5. There was trust among the research project team members | Der var tillid blandt forsker projektgruppens medlemmer | T5. Der var tillid blandt medlemmerne af forskningsprojektteamet | T5.  Der var tillid blandt forskningsprojektgruppens medlemmer | Der var tillid blandt medlemmerne i forskergruppen | There was an atmosphere of trust among the members of the research group | There was trust among the members of the research group |  | Der var tillid blandt medlemmerne i forsknings projekt gruppen | Jeg oplevede at der var tillid blandt medlemmerne af projektgruppen | Der var tillid blandt medlemmerne i forsknings projekt gruppen | Der var tillid blandt medlemmerne i forsknings projekt gruppen |

| **Support** | **Støtte** | **Støtte** | **Support** | **Støtte** | **Support** | **Support** | ens | **Støtte** |  |  | støtte |
| --- | --- | --- | --- | --- | --- | --- | --- | --- | --- | --- | --- |
| The following two (2) statements are about the support provided throughout the project. | De følgende to (2) udsagn omhandler den støtte, der gives igennem hele projektet. | De følgende to (2) udsagn handler om støtten, der blev givet gennem hele projektet. | De følgende to (2) udsagn handler om den støtte, der blev ydet gennem hele projektet. | De følgende to (2) udsagn handler om den støtte, der blev givet i forskningsprojektet. | The following two (2) statements relate to the support provided throughout the research project | The following two (2) statements are about the support that was given during the whole research project |  | De følgende to (2) udsagn handler om den støtte, der blev givet gennem hele projektet |  |  |  |
| SU1. I received sufficient support to contribute to the project (for example, orientation, readings, training workshops, webinars) | Jeg modtog tilstrækkelig støtte til at bidrage til projektet (for eksempel orientering, skriftligt materiale, træningsworkshops, webinarer) | SU1. Jeg fik tilstrækkelig støtte til at bidrage til projektet (f.eks. orientering, gennemlæsning af dokumenter, workshops, webinarer) | SU1.  Jeg modtog tilstrækkelig støtte til at bidrage til projektet (for eksempel information/orientering, læsning, træningsworkshops, webinarer) | Jeg modtog tilstrækkelig støtte til at kunne bidrage til forskningsprojektet (for eksempel information, introduktion, undervisning, workshops, webinarer) | I was given satisfactory support to be able to participate in the research project (e.g. information, teaching, workshops, webinars) | I received sufficient support to be able to contribute to the research project (for example information, teaching, workshops, webinars) | To be able to | Jeg modtog tilstrækkelig støtte til at kunne bidrage til forskningsprojektet (for eksempel mundtlig og skriftligt information, oplæring undervisning, workshops, webinarer) | Jeg modtog nok støtte til at kunne bidrage til projektet (for eksempel mundtlig og skriftlig information, oplæring undervisning, workshops, webinarer osv.) | Jeg modtog nok støtte til at kunne bidrage til projektet (for eksempel mundtlig og skriftlig information, oplæring undervisning, workshops, webinarer) | Jeg modtog nok støtte til at kunne bidrage til projektet (for eksempel mundtlig og skriftlig information, oplæring undervisning, workshops, webinarer osv.) |
| SU2.Any concerns I had were addressed | Mine eventuelle bekymringer blev italesat | SU2. Alle de spørgsmål, jeg havde, blev taget op. | SU2.  Eventuelle bekymringer, jeg havde, blev imødekommet | Eventuelle bekymringer, jeg havde, blev imødekommet | Any concerns I had were listened to | Any concerns I had were addressed |  | Eventuelle bekymringer, jeg havde, blev imødekommet | Der blev taget hånd om eventuelle udfordringer  Hvis jeg havde bekymringer, blev der taget  hånd om dem  Mine eventuelle bekymringer blev der taget hånd om | Hvis jeg havde bekymringer, blev der taget  hånd om dem | Hvis jeg havde bekymringer, blev der taget  hånd om dem |

| **Feel Valued** | **Føle sig værdsat** | **Om at føle sig værdsat** | **Føle sig værdsat** | **Føle sig værdsat** | **Appreciation** | **Feeling valued** |  | **Føle sig værdsat** |  |  |  |
| --- | --- | --- | --- | --- | --- | --- | --- | --- | --- | --- | --- |
| The following two (2) statements are about your feeling of being a valued member of the research team. | De følgende to udsagn omhandler din følelse af at være et værdsat medlem af forskerholdet. | De følgende to (2) udsagn handler om din følelse af at være et værdsat medlem af forskningsteamet. | De følgende to (2) udsagn handler om din følelse af at være et værdsat medlem af forskerteamet. | De følgende to (2) udsagn handler om din følelse af at være et værdsat medlem i forskergruppen | The following two (2) statements relate to your sense of being a valued member of the research group | The following two (2) statements are about your feeling of being a valued member of the research group |  | De følgende to (2) udsagn handler om din følelse af at være et værdsat medlem i forskergruppen |  |  |  |
| FV1.The research project team appreciated my contributions | Forsknings projektgruppen værdsatte mine bidrag | FV1. Forskningsprojektteamet satte pris på mine bidrag | FV1. Forskningsprojektgruppen satte pris på mine bidrag | Forskergruppen satte pris på mine bidrag | The research group appreciated my input | The research group appreciated my contributions |  | Forskergruppen satte pris på mine bidrag | Forskergruppensatte pris på mine meninger/input | Forskningsprojekt gruppen satte pris på mine bidrag |  |
| FV3.  I was offered sufficient recognition for my contributions (for example, payment, authorship, or gifts) | Jeg blev tilbudt tilstrækkelig anerkendelse for mine bidrag (f.eks. betaling, forfatterskab eller gaver) | FV3. Jeg blev tilbudt tilstrækkelig anerkendelse for mine bidrag (f.eks. betaling, forfatterskab eller gaver) | FV3.  Jeg blev tilbudt tilstrækkelig anerkendelse for mine bidrag (f.eks. betaling, forfatterskab eller gaver) | Jeg blev tilbudt tilstrækkelig anerkendelse for mine bidrag (f.eks. betaling, forfatterskab eller gaver) | I was given satisfactory recognition for my input (e.g. in the form of remuneration, authorship or gifts) | I was offered adequate recognition of my contributions (for example payment, authorship or gifts) |  | Jeg blev tilbudt tilstrækkelig anerkendelse for mine bidrag (f.eks. betaling, forfatterskab eller gaver) | Udelade betaling mv.  Jeg blev tilbudt tilstrækkelig anerkendelse for mine bidrag (f.eks. forplejning, forfatterskab, foredrag og gaver) | Jeg blev tilbudt tilstrækkelig anerkendelse for mine bidrag (f.eks. forplejning, forfatterskab, foredrag eller gaver) | Jeg blev tilbudt tilstrækkelig anerkendelse for mine bidrag (f.eks. forplejning, forfatterskab, foredrag eller gaver) |

| **Benefits** | **Fordele** | **Fordele** | **Fordele** | **Fordele** | **Benefits** | **Benefits** | ens | **Fordele** |  |  |  |
| --- | --- | --- | --- | --- | --- | --- | --- | --- | --- | --- | --- |
| The following three (3) statements are about the benefits of your involvement in the project. | De følgende tre udsagn omhandler fordelene af dit engagement i projektet. | De følgende tre (3) udsagn handler om fordelene ved din inddragelse gennem hele projektet. | De følgende tre (3) udsagn handler om fordelene ved din involvering i projektet. | De følgende tre (3) udsagn handler om fordelene ved din involvering i forskningsprojektet. | The following three (3) statements relate to how you benefited from participating in the research project | The following three (3) statements are about the benefits of your participation in the research project |  | De følgende tre (3) udsagn handler om fordelene ved din involvering i projektet |  |  |  |
| BE1.I enjoyed being a part of the project | Jeg nød at være en del af projektet | BE1. Jeg nød at være en del af projektet | BE1.  Jeg nød at være en del af projektet | Jeg nød at være en del af forskningsprojektet | I enjoyed being part of the research project | I enjoyed being a part of the research project | ens | Jeg nød at være en del af projektet | Jeg sætter pris på at have været en del af projektet  Nydelse ikke relevant her. | Jeg nød at være en del af projektet | Jeg nød at være en del af projektet |
| BE2. I made an impact on the decisions in the project | Jeg har haft indflydelse på beslutningerne i projektet | BE2. Jeg har haft indflydelse på beslutningerne i projektet | BE2.  Jeg fik indflydelse på beslutningerne i projektet | Jeg fik indflydelse på beslutningerne i forskningsprojektet | I had a say in decisions on the research project | I had influence on the decisions of the research project |  | Jeg fik indflydelse på beslutningerne i projektet | Jeg fik indflydelse på beslutningerne i projektet | Jeg fik indflydelse på beslutningerne i projektet | Jeg fik indflydelse på beslutningerne i projektet |
| BE4. My involvement had positive impacts on my life | Mit engagement havde en positiv indvirkning på mit liv | BE4. Min inddragelse havde en positiv indvirkning på mit liv | BE4.  Mit engagement havde en positiv indvirkning på mit liv | Min involvering i forskningsprojektet havde positiv indvirken på mit liv | My involvement in the research project has had a positive impact on my life | My participation in the research project had a positive influence on my life |  | Min involvering i projektet havde positiv indvirken i mit liv | Den er ikke god.  Min involvering i projektet var positiv for mig | Min involvering i projektet havde positiv betydning i mit liv | Min involvering i projektet havde positiv betydning i mit liv |
