# Supplementary material for: Patient Engagement in Research Scale (PEIRS-22): Danish translation, applicability, and user experiences
Source: Res Involv Engagem. 2023 Dec 7;9:115. doi: 10.1186/s40900-023-00526-2 (PMC10704757; doi:10.1186/s40900-023-00526-2)
Supplement: Supplementary file 3 — Additional file 3. The Danish version of PEIRS-22. [file 40900_2023_526_MOESM3_ESM.docx]

**
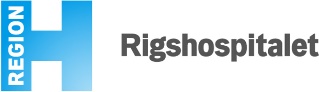
Involvering af patienter og pårørende i forskningsprojekter**

**Dansk version af PEIRS-22**

**Navn:**

**Dato/måned/år:**

**Titel på det projekt du deltager i:**

**Instruktioner:**

- Tænk på din oplevelse med at være involveret i projektet og en del af forskningsprojektgruppen.
- Sæt venligst kun ét kryds i boksen ved hvert udsagn.
- Hvis du er usikker på hvilket udsagn du skal vælge, så vælg det udsagn der falder dig først ind.
- Der er 22 spørgsmål og det vil tage dig cirka 3 til 7 minutter at besvare spørgeskemaet.

| **Generelt om projektet**  De følgende syv (7) udsagn handler om dine generelle oplevelser gennem hele projektet | | | | |
| --- | --- | --- | --- | --- |
| PR2. Forskningsprojektgruppens medlemmer blev ordentligt introduceret til hinanden | | | | |
| Meget enig | Enig | Neutral | Uenig | Meget uenig |
| PR9. Jeg havde generelt muligheder for at bidrage til projektet | | | | |
| Meget enig | Enig | Neutral | Uenig | Meget uenig |
| PR10. Jeg var i stand til at udføre mine opgaver i projektet | | | | |
| Meget enig | Enig | Neutral | Uenig | Meget uenig |
| PR11. Jeg var med til at træffe beslutninger om projektet | | | | |
| Meget enig | Enig | Neutral | Uenig | Meget uenig |
| PR12. Jeg modtog tilstrækkelige opdateringer om projektet | | | | |
| Meget enig | Enig | Neutral | Uenig | Meget uenig |
| PR13. Kommunikationen i forskningsprojektgruppen var tydelig gennem hele projektet | | | | |
| Meget enig | Enig | Neutral | Uenig | Meget uenig |
| PR14. Projektet var værd at bruge min tid på | | | | |
| Meget enig | Enig | Neutral | Uenig | Meget uenig |

| **Belejligt**  De følgende tre (3) udsagn handler om hvor belejligt det var for dig at bidrage gennem hele projektet | | | | |
| --- | --- | --- | --- | --- |
| CN1. Jeg havde mulighed for at være med til at vælge mine opgaver i projektet | | | | |
| Meget enig | Enig | Neutral | Uenig | Meget uenig |
| CN3. Jeg havde tilstrækkelig tid til at udføre mine opgaver gennem hele projektet | | | | |
| Meget enig | Enig | Neutral | Uenig | Meget uenig |
| CN4. Jeg havde mulighed for at udtrykke mine synspunkter | | | | |
| Meget enig | Enig | Neutral | Uenig | Meget uenig |

| **Bidrag**  De følgende tre (3) udsagn handler om dine bidrag gennem hele projektet | | | | |
| --- | --- | --- | --- | --- |
| CT1. Jeg bidrog til projektet ved at give mit perspektiv | | | | |
| Meget enig | Enig | Neutral | Uenig | Meget uenig |
| CT2. Det var en god brug af min tid at bidrage | | | | |
| Meget enig | Enig | Neutral | Uenig | Meget uenig |
| CT4. Mine opgaver i projektet var overskuelige | | | | |
| Meget enig | Enig | Neutral | Uenig | Meget uenig |

| **Samarbejde i forskningsprojektgruppen**  De følgende to (2) udsagn handler om samarbejdet i forskningsprojektgruppen gennem hele projektet | | | | |
| --- | --- | --- | --- | --- |
| T2. Jeg var en ligeværdig partner i forskningsprojektgruppen | | | | |
| Meget enig | Enig | Neutral | Uenig | Meget uenig |
| T5. Der var tillid blandt medlemmerne i forskningsprojektgruppen | | | | |
| Meget enig | Enig | Neutral | Uenig | Meget uenig |

| **Støtte**  De følgende to (2) udsagn, handler om den støtte der blev givet gennem hele projektet | | | | |
| --- | --- | --- | --- | --- |
| SU1. Jeg modtog nok støtte til at kunne bidrage til projektet (f.eks. mundtlig og skriftlig information, oplæring, undervisning, workshops, webinarer osv.) | | | | |
| Meget enig | Enig | Neutral | Uenig | Meget uenig |
| SU2. Hvis jeg havde bekymringer, blev der taget hånd om dem | | | | |
| Meget enig | Enig | Neutral | Uenig | Meget uenig |

| **Føle sig værdsat**  De følgende to (2) udsagn handler om din følelse af at være et værdsat medlem af forskningsprojektgruppen | | | | |
| --- | --- | --- | --- | --- |
| FV1. Forskningsprojekt gruppen satte pris på mine bidrag | | | | |
| Meget enig | Enig | Neutral | Uenig | Meget uenig |
| FV3. Jeg blev tilbudt tilstrækkelig anerkendelse for mine bidrag (f.eks. forplejning, forfatterskab, foredrag eller gaver) | | | | |
| Meget enig | Enig | Neutral | Uenig | Meget uenig |

| **Fordele**  De følgende tre (3) udsagn handler om fordelene ved din involvering i projektet | | | | |
| --- | --- | --- | --- | --- |
| BE1. Jeg nød at være en del af projektet | | | | |
| Meget enig | Enig | Neutral | Uenig | Meget uenig |
| BE2. Jeg fik indflydelse på beslutningerne i projektet | | | | |
| Meget enig | Enig | Neutral | Uenig | Meget uenig |
| BE4. Min involvering i projektet havde positiv betydning i mit liv | | | | |
| Meget enig | Enig | Neutral | Uenig | Meget uenig |

**Mange tak for din besvarelse**

**Reference:** Hamilton CB, Hoens AM, McKinnon AM, et al (2021) Shortening and validation of the Patient Engagement In Research Scale (PEIRS) for measuring meaningful patient and family caregiver engagement. Heal Expect 863–879.
