# Supplementary material for: Patient Engagement in Research Scale (PEIRS-22): Danish translation, applicability, and user experiences
Source: Res Involv Engagem. 2023 Dec 7;9:115. doi: 10.1186/s40900-023-00526-2 (PMC10704757; doi:10.1186/s40900-023-00526-2)
Supplement: Supplementary file 4 — Additional file 4. Guide for focus group interviews. [file 40900_2023_526_MOESM4_ESM.docx]

| **Topics** | **Questions** |
| --- | --- |
| **Procedural requirements** | When you think about your experience of being involved in the research project as a research partner, what do you believe has been particularly good? |
| **Convenience** | Do you feel the research group and the head of research have heard your opinions? |
| **Contributions** | What responsibilities/tasks have you been given? |
|  | Please explain what you think of these tasks? |
|  | Did the scope of the tasks correspond to the allocated time for discussion? |
|  | Please explain, what is especially important when the head of research presents a topic for discussion? |
|  | Do you want to be involved more/less, please explain? |
| **Team Environment and Interaction** | How would you characterize the atmosphere in the group? |
|  | Did you experience trust among the research team members? |
|  | What actions did the researcher take to put you at ease in the group? |
|  | Do believe anything else could have been done? |
| **Support** | Did you receive sufficient support to contribute to the research project (e.g., information, teaching, workshops, webinars) |
| **Feel valued** | How can research leaders best show you recognition for your work? |
|  | Were you offered sufficient recognition for your contributions (e.g. payment, authorship, or gifts) |
| **Benefits** | Please explain, how did you get involved in the research project and contact with the research leader? |
|  | Why where you interested in being involved in research? |
|  | What meaning does this have to you? |
|  | Did your involvement meet your expectations? |
| **About the PEIRS-22 questionnaire** | Do you have any comments on the layout of the questionnaire? |
|  | Do you believe anything was missing? |
|  | Is it clear what it's asking about? |
|  | Do you think this questionnaire provides a comprehensive picture of involvement in research projects? |
| **Evaluation** | Are you aware of anything else you think we should include? |
|  | What advice would you give to the next generation of researchers who are launching a patient advisory board? |
|  | Is there anything you think should have been done differently or better? |
|  | Any further comments? |
